# Decreased Skin-Mediated Detoxification Contributes to Oxidative Stress and Insulin Resistance

**DOI:** 10.1155/2012/128694

**Published:** 2012-08-01

**Authors:** Xing-Xing Liu, Chang-Bin Sun, Ting-Tong Yang, Da Li, Chun-Yan Li, Yan-Jie Tian, Ming Guo, Yu Cao, Shi-Sheng Zhou

**Affiliations:** ^1^Department of Physiology, Medical College, Dalian University, Dalian 116622, China; ^2^Department of Histology and Embryology, Medical College, Dalian University, Dalian 116622, China; ^3^Department of Pathology, Xinxiang Medical College, Xinxiang 453003, China; ^4^Department of Physiology, China Medical University, Shenyang 110001, China; ^5^College of Environmental and Chemical Engineering, Dalian University, Dalian 116622, China

## Abstract

The skin, the body's largest organ, plays an important role in the biotransformation/detoxification and elimination of xenobiotics and endogenous toxic substances, but its role in oxidative stress and insulin resistance is unclear. We investigated the relationship between skin detoxification and oxidative stress/insulin resistance by examining burn-induced changes in nicotinamide degradation. Rats were divided into four groups: sham-operated, sham-nicotinamide, burn, and burn-nicotinamide. Rats received an intraperitoneal glucose injection (2 g/kg) with (sham-nicotinamide and burn-nicotinamide groups) or without (sham-operated and burn groups) coadministration of nicotinamide (100 mg/kg). The results showed that the mRNA of all detoxification-related enzymes tested was detected in sham-operated skin but not in burned skin. The clearance of nicotinamide and *N* ^1^-methylnicotinamide in burned rats was significantly decreased compared with that in sham-operated rats. After glucose loading, burn group showed significantly higher plasma insulin levels with a lower muscle glycogen level than that of sham-operated and sham-nicotinamide groups, although there were no significant differences in blood glucose levels over time between groups. More profound changes in plasma H_2_O_2_ and insulin levels were observed in burn-nicotinamide group. It may be concluded that decreased skin detoxification may increase the risk for oxidative stress and insulin resistance.

## 1. Introduction

Oxidative stress is a condition of oxidant/antioxidant imbalance in which the net amount of reactive oxygen species (ROS) exceeds the antioxidant capacity of the body [1, 2]. Increasing evidence suggests that oxidative stress might play a causal role in insulin resistance (IR) which is characterized by hyperinsulinemia [1–3]. One of the major sources of ROS is xenobiotics, which are exogenous chemicals such as heavy metals, drugs, insecticides, and food additives [3–5]. Another major source of ROS is endogenous toxic and bioactive compounds, such as catecholamines, the major stress hormones inactivated by catechol-*O*-methyltransferase (COMT) and monoamine oxidase (MAO) [6]. Thus, the efficiency of detoxification and elimination of xenobiotics and endogenous toxic compounds should be a crucial factor in oxidative stress and IR.

Xenobiotics are detoxified by xenobiotic/drug-metabolizing enzymes [7–9]. Thus, it is conceivable that any tissues/organs that are involved in xenobiotics detoxification/excretion and ROS clearance may play a role in oxidative stress and IR. The skin, which is the largest organ of the body, can degrade, inactivate, and eliminate numerous xenobiotics and endogenous toxic compounds through its xenobiotic/drug-metabolizing enzymes [7–9], ROS-scavenging system [10], and sweat glands [11–14]. It has been found that severe burns, which induce permanent structural tissue damage, are associated with longlasting IR, endoplasmic reticulum stress response, hypermetabolism, and elevation of cortisol, catecholamines, and cytokines (i.e., they still persist after burns have healed) [15–17]. Moreover, impaired cutaneous vasodilation and sweating are found in grafted skin [18]. These lines of evidence raise the possibility that a decrease in the skin functions might play a role in the development of oxidative stress and IR. To test this hypothesis, the present study investigated the relationship between the skin detoxification and ROS generation and IR by examining burn-induced changes in the degradation and clearance of nicotinamide which is the precursor of nicotinamide-adenine dinucleotide and is known to induce IR [19, 20].

## 2. Materials and Methods

### 2.1. Animal Experiment

#### 2.1.1. Glucose and Nicotinamide Loading Test

Animal experiment was conducted in accordance with institutional guidelines. Male Sprague-Dawley rats (180–220 g) were fed a standard rat chow. A 40% total body surface area full-thickness injury was inflicted on the back skin of the rat; sham-operated rats were subjected to an identical procedure, except that they were immersed in 25°C water, as previously described [20]. Rats were randomized into four groups: sham-operated group (*n* = 8), sham-nicotinamide group (*n* = 8), burn group (*n* = 9), and burn-nicotinamide group (*n* = 9). At 48 hours after burn injury or sham treatment and 12 h after fasting, all rats in the four groups received intraperitoneal injection of glucose (2 g/kg body weight) with (sham-nicotinamide group and burn-nicotinamide group) or without (sham-operated group and burn group) coinjection of nicotinamide (Sigma, St. Louis, MO, USA; 100 mg/kg body weight). Tail blood glucose level was monitored before and at 15, 30, and 60 min after glucose injection using a glucometer (OneTouch Ultra, LifeScan, Milpitas, CA, USA). At the same timepoints, samples of tail blood (200 *μ*L) were collected for later determination of serum concentrations of insulin and hydrogen peroxide (H_2_O_2_). At the end of experiment (i.e., 1 h after glucose loading), blood sample was collected by eye bleed into EDTA tubes under urethane anesthesia (1.5 g/kg, ip); samples of liver, muscle (rectus femoris, biceps brachii, latissimus dorsi, and gastrocnemius), and back skin (in burned or sham-operated areas) were then harvested and stored in liquid nitrogen for later analysis. Plasma was separated by centrifugation (1500 g, 10 min) and stored at −80°C until assay.

#### 2.1.2. Reverse Transcription Polymerase Chain Reaction (RT-PCR) 

Total RNA of rat back skin and muscle tissues (rectus femoris, biceps brachii, and latissimus dorsi) was extracted using Trizol (Invitrogen, Carlsbad, CA, USA) according to the manufacturer's protocol. The mRNA of nicotinamide *N*-methyltransferase (NNMT), aldehyde oxidase (AOX1), COMT, MAO (monoamine oxidase A), superoxide dismutase 2, glutathione peroxidase 1, catalase (CAT), peroxiredoxin 1 (PRDX1), and neutrophil cytosolic factor 1 (p47-phox) was detected by RT-PCR. Extracted RNA was reverse-transcripted in a 20 *μ*L reaction with both oligo (dT) and random primers using PrimeScript RT Master Mix (Takara, Shiga, Japan). PCR amplification was performed in a Techne TC-512 gradient thermal cycler (Progene, Techne Ltd., Cambridge, UK), using specific primers listed in Table 1. PCR reaction conditions were as follows: 95°C for 10 min; 40 cycles of 95°C for 30 s, 60°C for 30 s, and 72°C for 45 s; followed by an extension reaction at 72°C for 10 min. The reaction products were analyzed by agarose gel electrophoresis and visualized by UV light after staining with ethidium bromide.

#### 2.1.3. Assays of Insulin, H_**2**_O_**2**_, and Glycogen

Serum insulin levels were determined with ELISA (Rat/Mouse Insulin Kit; Millipore, St. Charles, MO, USA). Serum H_2_O_2_ concentrations were measured using an H_2_O_2_ Assay Kit (Beyotime Biotechnology, Jiangsu, China). Hepatic and muscle (gastrocnemius) glycogen contents were determined with Glycogen Assay Kits (Nanjing Jiancheng Bioengineering Institute, Nanjing, China). ELISA plates were read by microplate reader (Bio-Rad, Hercules, CA, USA).

#### 2.1.4. Determination of Nicotinamide and *N* ^1^-Methylnicotinamide 

High-performance liquid chromatography (HPLC) was used to measure plasma nicotinamide and *N* ^1^-methylnicotinamide, as previously described [20]. HPLC system consisted of an LC-9A pump (Shimadzu, Kyoto, Japan), a Rheodyne 7725i sample injector with a 20-*μ*L sample loop (Rheodyne LLC, Rohnert Park, CA, USA), a Hypersil ODS C18 column (Thermo, Bellefonte, PA, USA) with a Waters 470 fluorescence detector (Milford, MA, USA). All chromatography was performed at room temperature.

### 2.2. Human Experiments

All patients participating in the study signed an informed consent form before the liver and skin samples for the immunohistochemistry analysis. Human normal liver and skin samples were donated by individuals who underwent resection of liver cancer and breast cancer, respectively. This study has been approved by the Ethics Committee of Dalian University.

#### 2.2.1. Immunohistochemistry 

Histological examination of the resected livers and skin ensured the use of healthy tissue. Samples of liver and skin were plunged directly into liquid nitrogen and subsequently stored at −80°C until assay. The location of AOX1 in human skin was determined by immunohistochemical staining. Briefly, four-micrometer sections were deparaffinized in xylene and rehydrated through graded ethanol. After microwave antigen retrieval, immunoreactivity was detected by streptavidin-biotin-peroxidase complex method (Fuzhou Maixin, Fuzhou, China) with color development using 3, 3′-diaminobenzidine. The primary antibody was mouse anti-AOX1 (1 : 300; BD Biosciences, CA, USA). Substituting phosphate-buffered saline (PBS) for the primary antibody was used as the negative control. Sections were counterstained with Mayer's hematoxylin for 30 s. Sections of normal hepatic tissue were used as a positive control.

#### 2.2.2. Western Blotting 

Western blotting analysis of NNMT and AOX1 was performed according to standard protocols. Briefly, 30 *μ*g human liver or skin protein was separated by 12% (for NNMT) and 8% (for AOX1) SDS polyacrylamide gels, respectively, and transferred to polyvinyl difluoride membranes (Millipore, MA, USA). The membranes were blocked in TBS containing 0.1% Tween-20 and 5% nonfat dry milk for 60 min at room temperature and incubated with antibody to NNMT (1 : 1000; Santa Cruz Biotechnology, CA, USA) and AOX1 (1 : 600; BD Biosciences, CA, USA) overnight at 4°C. Then, the membranes were washed by PBS-Tween followed by 1 h incubation at room temperature with horseradish peroxidase-conjugated secondary antibody (1 :  5000; Santa Cruz Biotechnology, CA, USA) and detected using the enhanced chemiluminescence (Amersham Life Science, NJ, USA). Human hepatic tissues were used as a positive control for the two antibodies.

### 2.3. Statistical Analysis 

The data are presented as means ± SEM. Statistical differences in the data were evaluated by Student's *t*-test or one-way ANOVA as appropriate and were considered significant at *P* < 0.05.

## 3. Results

### 3.1. Burn-Induced Changes in Gene Expression in Rat Skin 

**Figure 1:**
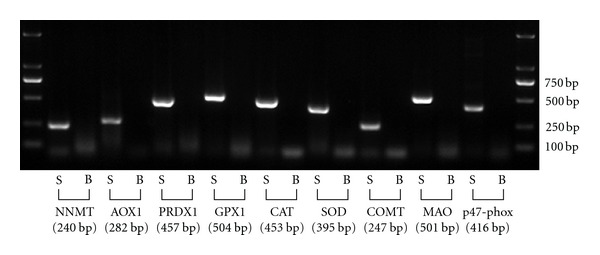
RT-PCR analysis of mRNA expression of xenobiotic/drug- and ROS-metabolizing enzymes in rat skin. S: sham-operated skin. B: burned skin. The data shown are representative of three separate experiments.

We first compared the gene expression of xenobiotic/drug-metabolizing enzymes (including NNMT, AOX1, COMT, and MAO), ROS-scavenging enzymes (including SOD, GPX1, CAT, and PRDX1), and p47-phox, a component of the NADPH oxidase complex, between sham-operated rat skin and burned rat skin. The RT-PCR results showed that all of the enzymes tested were expressed in sham-operated rat skin, but there was no detectable expression of the enzymes in burned skin (Figure 1). These results suggest that full-thickness burn may lead to loss of the detoxification and antioxidant functions of the skin.

### 3.2. Burn-Induced Changes in the Degradation and Clearance of Nicotinamide

**Figure 2:**
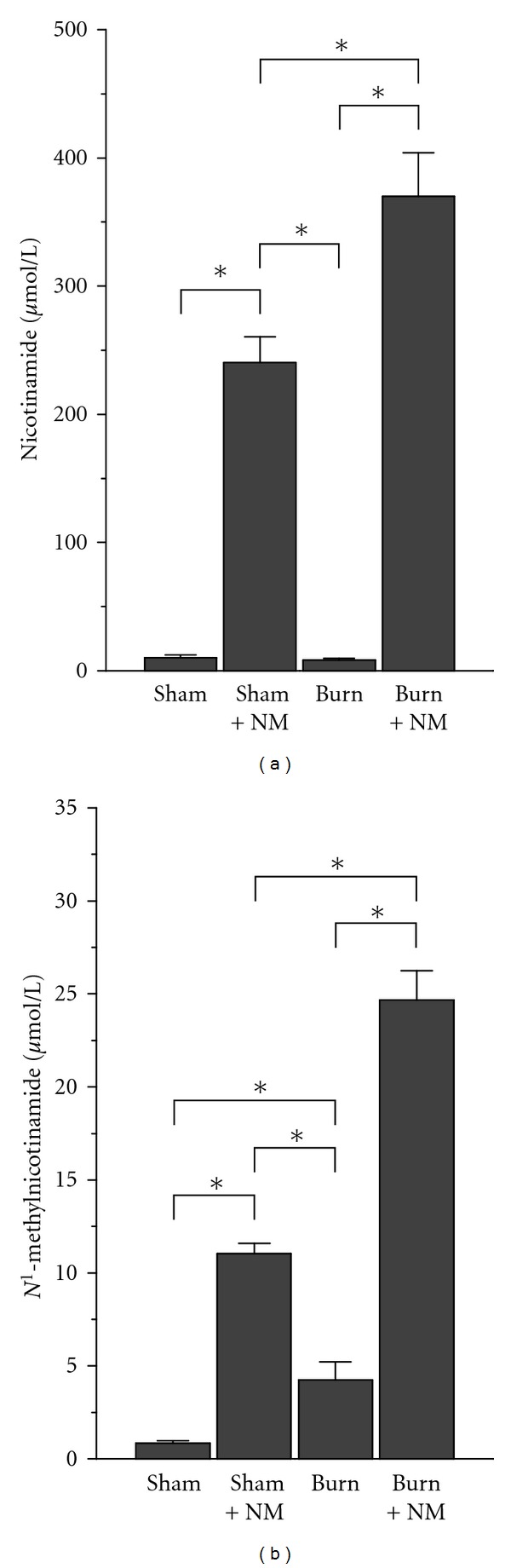
Plasma concentrations of nicotinamide and *N* ^1^-methylnicotinamide in different rat groups. Sham: sham-operated group; Sham + NM: sham-nicotinamide group; Burn: burn group; Burn + NM: burn-nicotinamide group. *P < 0.01. Bar graph indicates mean ± SEM.

The observations that rat skin expresses nicotinamide-degrading enzymes suggest that the skin may play a role in nicotinamide clearance. Therefore, we investigated the effect of burn on the levels of plasma nicotinamide and *N* ^1^-methylnicotinamide, the toxic-methylated metabolite of nicotinamide [20]. Sham-nicotinamide group and burn-nicotinamide group showed significantly higher plasma nicotinamide levels compared with the other two groups, and the level of plasma nicotinamide in burn-nicotinamide group was significantly higher than that of sham-nicotinamide group (Figure 2(a)). These results suggest that the tolerance of burned rats to nicotinamide is decreased. Although there was no significant difference in plasma nicotinamide levels between sham-operated group and burn group (Figure 2(a)), the plasma *N* ^1^-methylnicotinamide level in burn group was much higher than that of sham-operated group (Figure 2(b)). Burn-nicotinamide group showed a significantly higher plasma level of *N* ^1^-methylnicotinamide than other groups (Figure 2(b)). These results indicate that burn decreases the clearance of *N* ^1^-methylnicotinamide.

### 3.3. Burn Enhances the H_**2**_O_**2**_-Generating and IR-Inducing Effects of Nicotinamide

Nicotinamide is known to induce oxidative stress, IR and glucose intolerance [19–21]. We therefore, examined the responses of rats of different groups to glucose load.

The results showed that there were no significant differences in the baseline serum concentration of H_2_O_2_ (a major component of ROS) between the groups.

**Figure 3:**
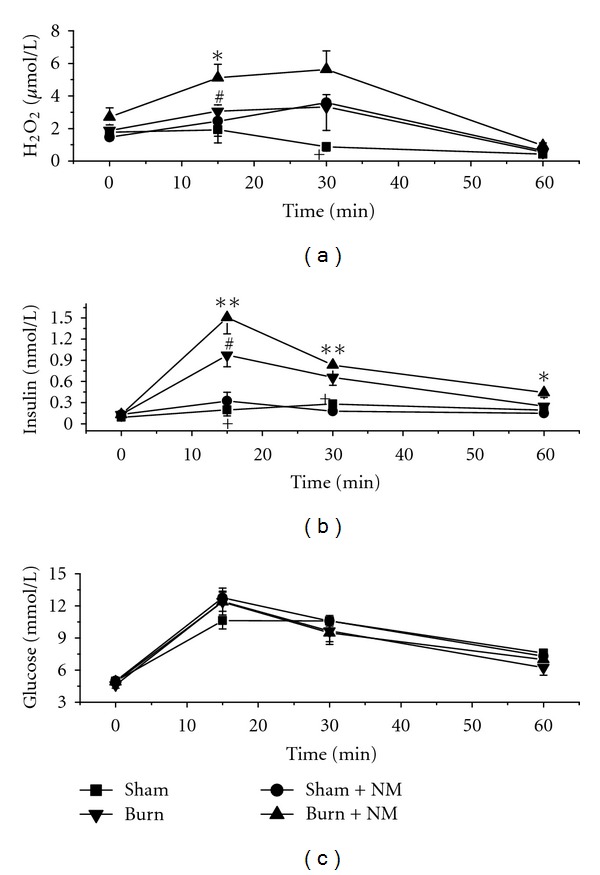
Serum H_2_O_2_ and insulin levels and blood glucose concentrations in different rat groups. Sham: sham-operated group; Sham + NM: sham-nicotinamide group; Burn: burn group; Burn + NM: burn-nicotinamide group. *P < 0.05 and **P < 0.01 versus sham-nicotinamide group, ^#^ *P* < 0.05 versus burn-nicotinamide group and ^+^ *P* < 0.01 versus burn group at the same time point. Data were presented as means ± SEM.

Glucose load induced a decline in the plasma H_2_O_2_ level in sham-operated group, while the other three groups showed an increasing trend. The increasing trend was more profound in burn-nicotinamide group than in sham-nicotinamide group and burn group (Figure 3(a)).

The plasma insulin levels showed increasing trends in all groups with a peak at 15 or 30 min after glucose loading. The changes in plasma insulin were more profound in burn-nicotinamide group and burn group (Figure 3(b)), whereas there were no significant differences in the changing trends in blood glucose levels between groups (Figure 3(c)). These results indicate that burn can induce IR, which can be further enhanced by nicotinamide load.

**Figure 4:**
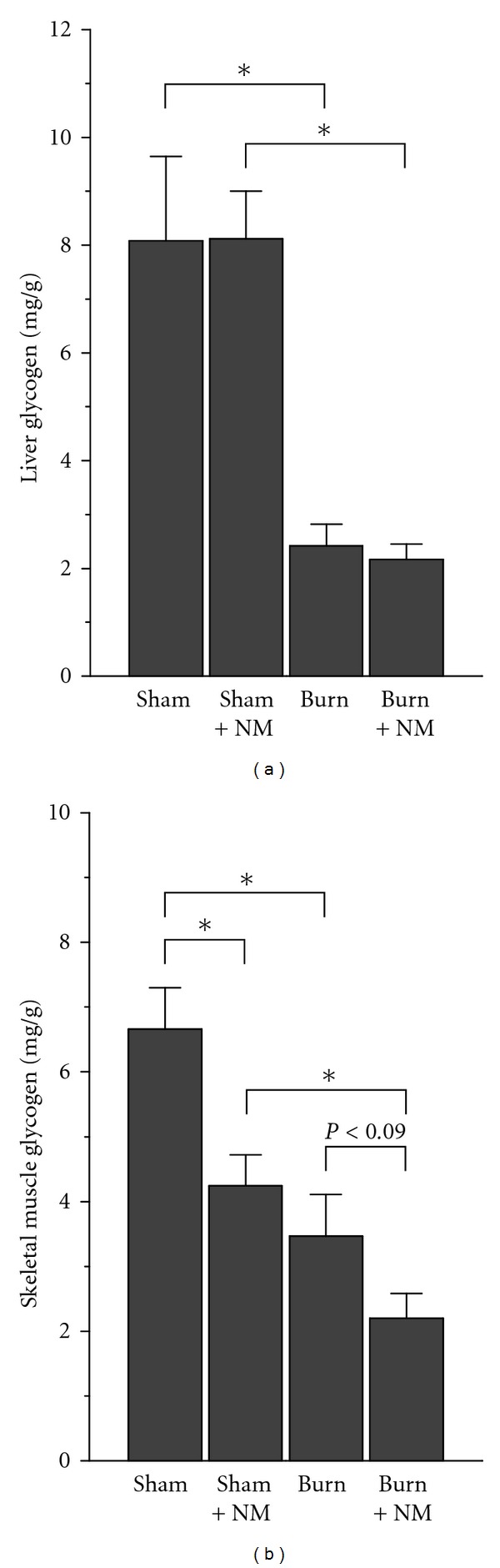
Glycogen contents of liver and skeletal muscle in different rat groups. Sham: sham-operated group; Sham + NM: sham-nicotinamide group; Burn: burn group; Burn + NM: burn-nicotinamide group. *P < 0.01. Bar graph indicates mean ± SEM.

There were no significant differences in liver glycogen contents either between sham-operated group and sham-nicotinamide group or between burn group and burn-nicotinamide group. However, the two burn groups showed significantly lower liver glycogen contents than that of the two sham groups (Figure 4(a)). It seems that the liver glycogen synthesis is affected by burn, but not by nicotinamide load. In contrast, the muscle glycogen levels were affected by both burn and nicotinamide load (Figure 4(b)).

**Figure 5:**
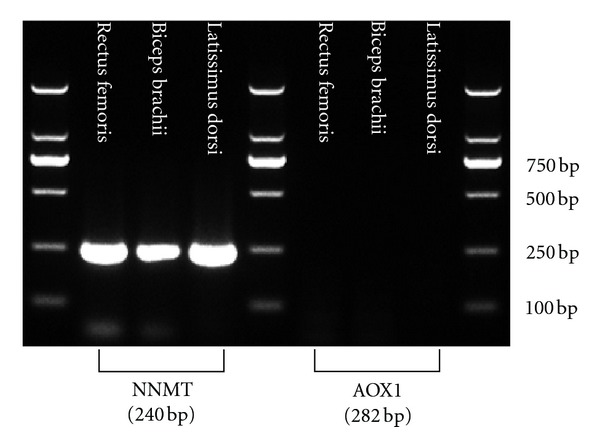
RT-PCR analysis of mRNA expression of NNMT and AOX1 in the muscle from sham-operated rat. The data shown are representative of three separate experiments.

To explore the mechanism underlying these different responses of liver and skeletal muscle to nicotinamide load, we detected the expression of NNMT and AOX1 in rat skeletal muscle. The RT-PCR results showed that rat skeletal muscle expressed NNMT, but did not express AOX1 (Figure 5). This suggests that the skeletal muscle could convert nicotinamide to *N* ^1^-methylnicotinamide, but could not detoxify *N* ^1^-methylnicotinamide.

### 3.4. Expression of NNMT and AOX1 in Human Skin

**Figure 6:**
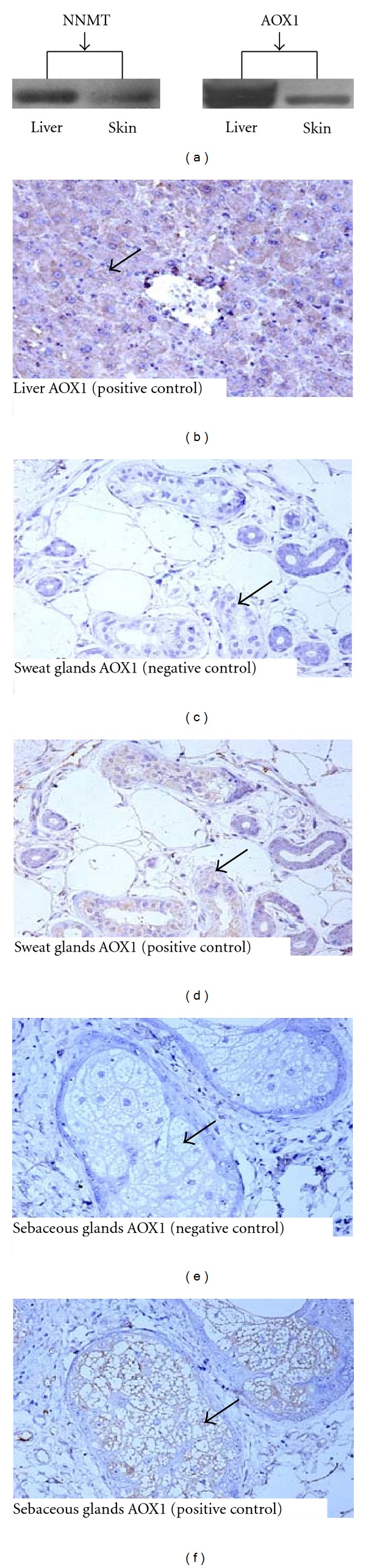
Expression of NNMT and location of AOX1 in human liver and skin. (a) western blotting results. ((b)–(f)) immunohistochemistry analysis of AOX1 expression. (b) positive control of AOX1 expression (human liver). ((d) and (f)), showing the location of AOX1 in sweat glands and sebaceous glands, respectively. ((c) and (e)) negative control for ((d) and (f)) respectively (without primary antibody). AOX1 was stained using a streptavidin-biotin-peroxidase complex method with diaminobenzidine substrate. Nuclei were counterstained with hematoxylin. Magnification: ×200.

We then detected the expression of the NNMT and AOX1 in human skin. The results of western blotting showed that both NNMT and AOX1 were expressed in the human skin (Figure 6(a)). Immunohistochemical analysis showed that AOX1 was located in sweat glands (Figure 6(d)) and sebaceous glands (Figure 6(f)). These results suggest that human skin, like rat skin, may also be involved in the degradation of excessive nicotinamide.

## 4. Discussion

The main results of the present study are that: (1) the expression of xenobiotic/drug-metabolizing enzymes and ROS-scavenging enzymes was not detected in the full-thickness burned rat skin; (2) burn could decrease the clearance of excessive nicotinamide in rats, which was accompanied by increased H_2_O_2_ generation and abnormal response to glucose load, that is, elevated insulin levels and decreased glycogen levels in skeletal muscle; (3) rat skeletal muscle was found to express NNMT, but not AOX1; (4) the expression of NNMT and AOX1 was detected in human skin.

Excessive nicotinamide is primarily degraded to *N* ^1^-methylnicotinamide by NNMT and then further oxidized to pyridones by AOX1 [20]. Thus, it is expected that any tissues/organs that express NNMT and AOX1 may contribute to the body's total capacity to degrade excessive nicotinamide. The skin expresses NNMT, thus, burn-induced increase in the level of plasma nicotinamide might be due to a change in skin-mediated nicotinamide degradation. Plasma *N* ^1^-methylnicotinamide levels are determined not only by its generation from nicotinamide, but also by its conversion to pyridones. NNMT is widely expressed in human and rat tissues [22, 23], especially in skeletal muscle, while AOX1 is not expressed in the skeletal muscle (Figure 5). Skeletal muscle is the largest tissue in vertebrates. Obviously, the uninvolvement of skeletal muscle in *N* ^1^-methylnicotinamide degradation increases the importance of skin contribution in the conversion, which may account for nicotinamide load-induced more profound increase in plasma *N* ^1^-methylnicotinamide level in burned rats than in sham-operated rats.

Evidence suggests that IR may be the consequence of oxidative stress [1–3]. Indeed, our previous study has shown that coadministration of glucose and nicotinamide can induce IR due to excessive ROS generation caused by nicotinamide degradation in human subjects [21]. The data from the present study showed that decreased skin-mediated nicotinamide degradation could enhance the ROS generating and IR-inducing effects of nicotinamide. *N* ^1^-methylnicotinamide is a toxic intermediate of nicotinamide metabolism. The liver not only degrades nicotinamide to *N* ^1^-methylnicotinamide, but also further detoxifies *N* ^1^-methylnicotinamide to pyridones. Unlike the liver, the skeletal muscle can only convert nicotinamide to *N* ^1^-methylnicotinamide. This may thus lead to an accumulation of *N* ^1^-methylnicotinamide in muscle tissue and thus cause a local toxic effect, which may explain why IR after nicotinamide load only occurs in the skeletal muscle, but not in the liver. The finding that burn alone decreased liver glycogen contents may involve other mechanisms that are beyond the scope of the present study.

It should be noted that besides nicotinamide-degrading enzymes, other xenobiotic/drug-metabolizing enzymes, such as cytochromes P450, flavin monooxygenases, MAO, COMT, glutathione-*S*-transferases, *N*-acetyltransferases, and sulfotransferases, are also expressed in the skin [7–9]. Numerous xenobiotics and endogenous bioactive/toxic substances are the substrates of skin xenobiotic/drug-metabolizing enzymes. The skin also expresses SOD, CAT, GPX1, and PRDX1, which can remove ROS, and thus help protect against oxidative damage [10, 24]. Moreover, human eccrine sweat glands, which total roughly one kidney, that is, 100 g, can excrete numerous xenobiotics, such as, metals and drugs [11, 13], and endogenous bioactive substances, such as, neurotransmitters, cytokines, and sterols [13, 25, 26]. Thus, it is conceivable that decreased skin function (e.g., due to burn injuries and cold ambient temperature) may increase the risk of an accumulation of toxic substances in the body and subsequent oxidative stress and IR.

Whether or not oxidative stress occurs not only depends on ROS production, but also on the body's total antioxidant capacity [1, 2]. The latter represents a sum of the antioxidant capacity of various organs. Each organ/tissue makes a distinctive contribution to the body's total antioxidant system. For example, the liver is mainly responsible for the biotransformation/detoxification of xenobiotics and endogenous toxic substances, while the kidney is mainly responsible for the elimination of toxic substances. Thus, a decrease in the functions of these organs is expected to increase the risk for oxidative stress and IR. Indeed, numerous studies have shown that patients with severe liver or kidney diseases are associated with oxidative stress and IR [27, 28]. The skin, like the liver and the kidneys, is one of the major contributors to the body's total antioxidant defense. Evidence has shown that (1) xenobiotics and endogenous toxic substances induce oxidative stress and IR [3]; (2) the skin is the largest organ involved in both the detoxification and excretion of xenobiotics and endogenous toxic substances [29]; (3) sauna, which increases the elimination of toxic substances [30], can improve cardiovascular, autoimmune, toxicant-induced, and other chronic health problems [25]; (4) there are sustained impairments in cutaneous vasodilation and sweating in grafted skin [18]; (5) there is an accumulation of endogenous toxic substances (e.g., cortisol, catecholamines, and cytokines) in the circulation of postburn patients [17]; (6) there is a decrease in the skin-mediated biotransformation of xenobiotics, as shown by the present study; (7) post-burn patients are associated with persistent endoplasmic reticulum stress and IR [15, 17]. Thus, post-burn oxidative stress and IR might involve decreased skin detoxification and excretion.

The above hypothesis is also supported by the observations in nonburn trauma and surgery. Both burn trauma and nonburn trauma (including major surgery), which are accompanied with increased sympathetic activity and redistribution of blood flow away from certain nonvital organs, such as the skin, gastrointestinal tract, and kidneys [31], are associated with IR during the acute phase. However, unlike burn trauma, the IR induced by nonburn trauma and surgery is transient and disappears after nonburn trauma or surgery [32], suggesting the following relationship: a permanent decrease in the skin function is associated with sustained IR, while a transient decrease in the function of intact skin is associated with transient IR. Moreover, the observation that there is an elevation in IR-inducing substances [33] and blood pressure [34] and worse symptoms of IR-related diseases [35, 36] in winter might also imply a close association between the skin function and IR, since low ambient temperature exposure reduces the blood flow to the skin and the skin temperature [37].

**Figure 7:**
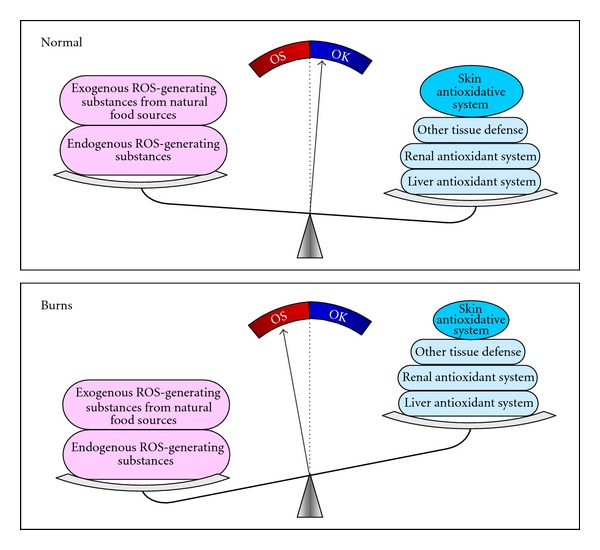
Proposed role of skin in oxidative stress and IR. The body's total antioxidant capacity, consisting of biotransformation system, excretion system, and reactive oxygen species clearance system, depends on the functions of body's tissues/organs. A decrease in the skin contribution, such as induced by severe burn and cold ambient environment, decreases the body's total antioxidant capacity and thus increases the risk for oxidative stress and subsequent IR. OK: the body's total antioxidant capacity > ROS generation; OS: oxidative stress, that is, ROS generation > the body's total antioxidant capacity.

In conclusion, the skin is one of the major components of the body's antioxidant defense system. Factors that decrease the skin function may lead to a decrease in the clearance of xenobiotics and endogenous toxic substances and subsequent increase in ROS generation, which may contribute to the development of IR. The proposed role of the skin in oxidative stress and IR is summarized in Figure 7.

## Figures and Tables

**Table 1:** Primer oligonucleotide sequences of selected genes.

Molecule (gene)		Primers
NNMT	Forward	5^′^-CAGAGCTGAGACACGATGGA
	Reverse	5^′^-GCAGGCAGAGAGAAGCTGAT
AOX1	Forward	5^′^-GTCCAGAAGCTTCCAGA
	Reverse	5^′^-GATGTTCACTGAGACCAAGA
COMT	Forward	5^′^-CTACTCAGCAGTGCGAATGG
	Reverse	5^′^-AAGTGTGTCTGGAAGGTAGCG
MAO	Forward	5^′^-GTGGCTCTTCTCTGCTTTGT
	Reverse	5^′^-AGTGCCAAGGGTAGTGTGTATCA
SOD	Forward	5^′^-CTCCCTGACCTGCCTTACGACT
	Reverse	5^′^-AAGCGACCTTGCTCCTTATTG
GPX1	Forward	5^′^-TCCACCGTGTATGCCTTCTCC
	Reverse	5^′^-CCTGCTGTATCTGCGCACTGGA
CAT	Forward	5^′^-GAGGCAGTGTACTGCAAGTTCC
	Reverse	5^′^-GGGACAGTTCACAGGTATCTGC
PRDX1	Forward	5^′^-GTGGATTCTCACTTCTGTCATCT
	Reverse	5^′^-GGCTTATCTGGAATCACACCACG
p47-phox	Forward	5^′^-AGCTCCCAGGTGGTATGATG
	Reverse	5^′^-TGTCAAGGGGCTCCAAAT
